# THOC5, a member of the mRNA export complex: a novel link between mRNA export machinery and signal transduction pathways in cell proliferation and differentiation

**DOI:** 10.1186/1478-811X-12-3

**Published:** 2014-01-10

**Authors:** Doan D H Tran, Alexandra Koch, Teruko Tamura

**Affiliations:** 1 Institut fuer Biochemie, OE4310, Medizinische Hochschule Hannover, Carl-Neuberg-Str. 1, D-30623, Hannover, Germany

**Keywords:** mRNA export protein THOC5, Immediate-early gene response, Signal transduction, Cell differentiation

## Abstract

Cell growth, differentiation, and commitment to a restricted lineage are guided by a timely expressed set of growth factor/cytokine receptors and their down-stream transcription factor genes. Transcriptional control mechanisms of gene expression during differentiation have been mainly studied by focusing on the *cis*- and *trans-*elements in promoters however, the role of mRNA export machinery during differentiation has not been adequately examined. THO (Suppressors of the **t**ranscriptional defects of **h**pr1 delta by **o**verexpression) **c**omplex 5 (THOC5) is a member of THO complex which is a subcomplex of the **tr**anscription/**ex**port complex (TREX). THOC5 is evolutionarily conserved in higher eukaryotes, however the exact roles of THOC5 in transcription and mRNA export are still unclear. In this review, we focus on recently uncovered aspects of the role of THOC5 in signal transduction induced by extracellular stimuli. THOC5 is phosphorylated by several protein kinases at multiple residues upon extracellular stimuli. These include stimulation with growth factors/cytokines/chemokines, or DNA damage reagents. Furthermore, THOC5 is a substrate for several oncogenic tyrosine kinases, suggesting that THOC5 may be involved in cancer development. Recent THOC5 knockout mouse data reveal that THOC5 is an essential element in the maintenance of stem cells and growth factor/cytokine-mediated differentiation/proliferation. Furthermore, depletion of THOC5 influences less than 1% of total mRNA export in the steady state, however it influences more than 90% of growth factor/cytokine induced genes. THOC5, thereby contributes to the 3′ processing and/or export of immediate-early genes induced by extracellular stimuli. These studies bring new insight into the link between the mRNA export complex and immediate-early gene response. The data from these studies also suggest that THOC5 may be a useful tool for studying stem cell biology, for modifying the differentiation processes and for cancer therapy.

## Introduction

Cell lineage commitment and subsequent differentiation are tightly regulated by timely expressed cytokines/growth factors and their receptors. The binding of growth factors/cytokines to the corresponding receptor causes receptor activation and triggers the expression of different sets of immediate-early response genes, such as *v-ets erythroblastosis virus E26 oncogene homolog* (*Ets)* family genes, *Myc, Fos, early growth response 1 (Egr1)* or *JunB*. Transcriptional control mechanisms of the expression of these genes during differentiation were mainly studied by focusing on the *cis*- and *trans-*elements in promoters. However, recent findings reveal that the immediate-early gene response is also regulated by RNA processing machinery.

The THO complex, a sub-member of TREX (transcription/export), was originally identified in *Saccharomyces cerevisiae* as a five protein complex (Tho2p, Hpr1p, Mft1p, Thp2p, and Tex1) [1-6] that plays a role in transcriptional elongation, nuclear RNA export and genome stability. In higher eukaryotes such as *Drosophila melanogaster*[7] or humans [8], three proteins, (THOC1/hHpr1/p84, THOC2/hRlr1, and THOC3) and three additional unique proteins were identified, namely THOC5/Fms interacting protein (FMIP) [9], THOC6 and THOC7, as members of the THO complex. In this review we will focus on the role of THOC5 in extracellular stimuli mediated signal transduction.

## THOC5 is conserved from *Drosophila melanogaster* to *human*

**Figure 1:**
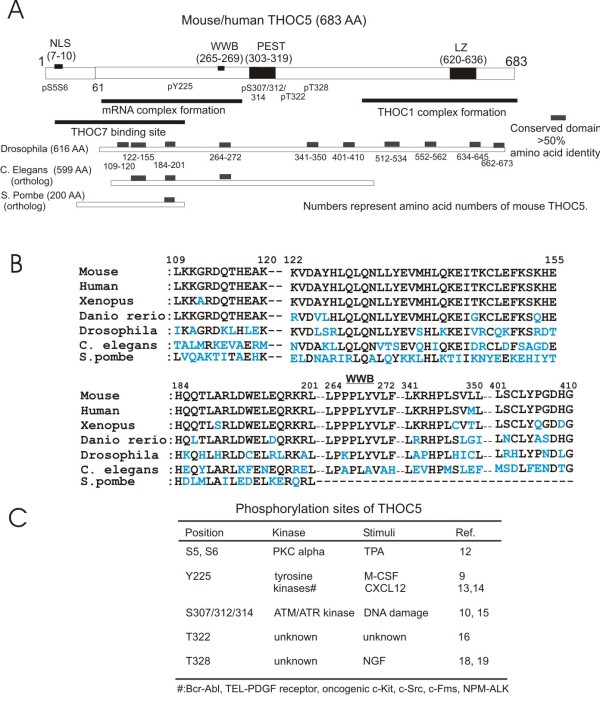
**Illustration of domain structure and phosphorylation sites of THOC5. A:** Arrangement of potential functional domains in THOC5. NLS: nuclear localization signal; WWB: potential binding site for proteins with WW-domains; PEST: potential PEST domain; LZ: potential leucine zipper; p: phosphorylation sites. Conserved domain (more than 50% amino acid identity in a 10 amino acids or longer stretch) between mouse THOC5 (683 amino acids long: 683AA) and *Drosophila melanogaster (Drosophila)* (616AA)*, C. elegans* (599AA) or *S. pombe* (200 AA) THOC5 is shown as grey boxes. Numbers represent amino acid numbers from mouse THOC5. **B**: An alignment of the key regions among mouse, human, *Xenopus tropicalis (Xenopus)*, *Danio rerio*, *Drosophila, C.elegans* and *S. pombe.***C**: Summary table of phosphorylation sites of THOC5.

Mouse and human THOC5 are 683 amino acids long. Amino acid identity of whole protein between human (NP_001002878.1) and mouse (NP_766026.1) is more than 96%, between mouse and *Xenopus tropicalis* (NP_001016827.1: 678 amino acids long) is 78%, between mouse and *Danio rerio* (AAH66736.1: 684 amino acids long) is 68%, and between mouse and *Drosophila melanogaster* (AAF47114.2: 616 amino acids long) is only 30%. However *Drosophila* THOC5 contains multiple short conserved sequences all over the molecule (Figure 1A,B). These include a potential WW domain binding site that may be engaged in protein/protein interaction (Figure 1A,B, WWB). Furthermore, THOC5 orthologs from *Caenorhabditis elegans* (*C. elegans*, CCD73969.1: 599 amino acids long) and *Schizosaccharomyces pombe* (*S. pombe*, CAB54812.1: 200 amino acids long) also contain three and one conserved sequences at the N-terminal domain, respectively (Figure 1A,B). Mouse, human and *Xenopus,* but not *Drosophila* or *C. elegans* THOC5 contains a PEST like domain, i.e. target sequence for proteolytic degradation. Furthermore, the human and mouse N-terminal domain (60–289 amino acid number) participates in forming a complex with mRNA [10] and also binds to THOC7, another member of the THO complex [11]. The C-terminal domain (500–683) is involved in the association with THOC1 [11] (Figure 1A).

## THOC5 is phosphorylated by extracellular stimuli

THOC5 is phosphorylated at multiple positions by extracellular stimuli, such as stimulation with growth factors/cytokines/chemokines, or DNA damage. The phosphorylation sites of THOC5 are summarized as following (see also Figure 1C).

Serine 5/6: Protein kinase C phosphorylates and inhibits nuclear import of THOC5 [12].

Tyrosine 225: It has been shown that THOC5 is a substrate for several tyrosine kinases such as macrophage-colony stimulating factor (M-CSF, or CSF-1) receptor, Fms [9], and various leukemogenic tyrosine kinases, such as Bcr-Abl (breakpoint cluster region-Abl tyrosine kinase fusion protein), translocation-ets-leukemia (TEL)-platelet derived growth factor (PDGF) receptor, oncogenic c-Kit (receptor for stem cell factor) or nucleophosmin (NPM)-anaplastic lymphoma kinase (ALK) [13,14]. Recent data showed that upon stimulation with chemokine (C-X-C motif) ligand 12 (CXCL12), tyrosine 225 is also phosphorylated via c-Src [13]. Notably, it has been clearly demonstrated that THOC5 is highly phosphorylated in CD34 positive chronic myeloid leukemia cells. Thus, tyrosine phosphorylation of THOC5 may be involved in leukemic cell behavior [13], however the biological consequence of tyrosine phosphorylation of THOC5 during leukemia development remains to be studied.

Serine 307/312/314: Three serine residues (S307/312/314) in the PEST domain of THOC5 have been shown to be directly phosphorylated by Ataxia-telangiectasia-mutated (ATM) kinase [15] followed by loss of the RNA binding potential of THOC5 [10], suggesting a novel connection between DNA damage and RNA export machinery. Notably, these serines are highly phosphorylated in testis [16] where ATM kinase plays a key role in spermatogenesis [17].

Threonine 322: this residue is phosphorylated in kidney, liver, spleen and testis by an unknown kinase [16].

Threonine 328: This phosphorylation was first reported using Akt kinase substrate antibody [18,19]. Upon stimulation with nerve growth factor (NGF), but not with insulin [18,19], threonine 328 is phosphorylated. Although ribosomal S6 kinase (RSK) and proto-oncogene kinase Pim1 phosphorylate at this residue *in vitro* (Koch and Tamura, unpublished data), it is not clear which protein kinase directly phosphorylates this residue *in vivo*.

Notably, all phosphorylation sites listed above are conserved among vertebrates, such as mouse, human, *Danio rerio*, *Latimeria chalumnae* (XP_006008521), *Geospiza fortis* (XP_005427642), and *Xenopus tropicalis* THOC5, but a corresponding amino acid was not found in *Drosophila melanogaster, C. elegans* or *S. pombe* THOC5.

## Subcellular localization of THOC5

**Figure 2:**
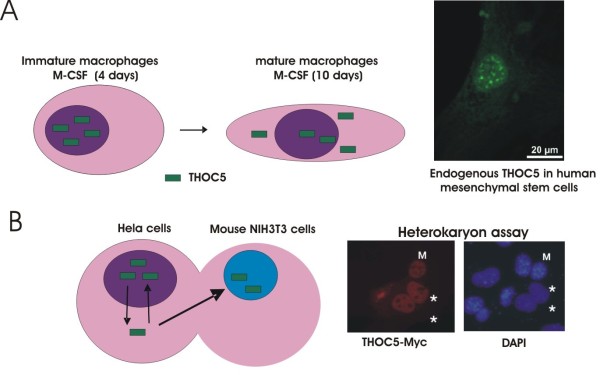
**Subcellular localization of THOC5. A**: Schematic representation of subcellular localization and immunostaining of endogenous THOC5. In immature macrophages, THOC5 is located mainly in the nucleus (nuclear speckle-like domains), while in mature macrophages THOC5 is also found in the cytoplasm [20]. Immunostaining: Human mesenchymal stem cells were fixed by methanol and then used for THOC5 specific staining using THOC5 monoclonal antibody [22]. THOC5 is located mainly in the nuclear speckles. **B**: Nuclear/cytoplasmic shuttling of THOC5 in Hela/mouse 3 T3 cells. Heterokaryon assay: myc-tagged THOC5 was expressed transiently in Hela cells which were then fused with mouse NIH/3 T3 cells in the presence of cycloheximide. Cells were stained by anti Myc-antibody followed by tetramethylrhodamine isothiocyanate (TRITC)-conjugated anti mouse IgG and with 4′,6-diamidino-2-phenylindole (DAPI). *: fused Hela cells; M: mouse NIH /3 T3 cells [23].

THOC5 is ubiquitously expressed in all organs [9]. It is located mainly in the nuclear speckles in bone marrow derived immature macrophages [20] or human mesenchymal stem cells (Figure 2A). In the terminal differentiated macrophages, however THOC5 is detected mainly in the cytoplasm [20], while in differentiated gut epithelial cells THOC5 is still in the nucleus [21]. This difference may be due to the quick turnover of gut epithelial cells.

Although THOC5 is detected mainly in the nucleus in NIH3T3 and Hela cells, heterokaryon assay experiment reveals that THOC5 is able to shuttle from human Hela cells to mouse NIH3T3 cells (Figure 2B), indicating that THOC5 is nuclear/cytoplasmic shuttling protein. It remains unknown whether THOC5 plays any role in the cytoplasm.

## THOC5 contributes to the differentiation process *in vivo* and *in vitro*

Earlier studies showed that the ectopic expression or the partial depletion of THOC5 in mouse myeloid progenitor or mesenchymal progenitor cell lines causes abnormal hematopoiesis or abnormal muscle differentiation, respectively, suggesting that the expression level of THOC5 is important for the normal differentiation process [9,14,22,24]. The depletion of THOC5 in mouse embryonic fibroblasts inhibits cell growth, and in bone marrow derived macrophages suppresses proliferation/differentiation, but did not result in apoptosis in either of these cases [20,25]. Along the same lines, *in vivo* studies using THOC5 knockout mice show that THOC5 is required for the maintenance of primitive cells, and the cell proliferation/differentiation. Notably, the depletion of THOC5 causes apoptosis of primitive cells, but not differentiated cells. We show that: firstly, conventional THOC5 knockout mice die at an early embryonic stage [26]. Secondly, in interferon inducible THOC5 knockout mice, the depletion of THOC5 causes rapid apoptosis of hematopoietic stem cells [26]. Thirdly, in tamoxifen inducible THOC5 knockout mice, the depletion of THOC5 impaired not only hematopoietic differentiation, but also differentiation and self-renewal of the whole gut epithelium. In this system although THOC5 depletion inhibits proliferation of cells in whole crypt domains, only few apoptotic cells were observed in the crypt domain [21]. Thus, THOC5 controls differentiation in a wide range of regenerative organs. On the other hand, depletion of THOC5 in terminally differentiated organs such as liver or kidney did not result in any pathological alterations [21]. Although hepatocytes express THOC5 at a high level [9], the mice in which THOC5 is depleted only in the liver survived for more than 3 months without any symptoms [26]. It has been shown that the conventional THOC1 (another member of THO complex) knockout mice are embryonic lethal [27]. However, using the tamoxifen inducible knockout mice system, knockout phenotype is quite different between THOC5 [21] and THOC1 [28]. Upon depletion of THOC1, pathological alteration was observed exclusively in stem cells from the small intestine, not in stem cells from colon, mammary gland or hematopoietic cells [28]. It is not clear whether the function of THOC1 and/or the efficiency of the tamoxifen inducible system are different than those of THOC5. Since THOC1 is also expressed ubiquitously the molecular mechanism which leads to alteration exclusively in small intestinal stem cells upon depletion of THOC1 remains to be studied.

## THOC5 dependent mRNAs

As described above, the depletion of THOC5 in liver does not result in a pathological phenotype. Furthermore, *albumin* or *transferrin* mRNAs were exported into the cytoplasm in the THOC5 depleted liver [21], indicating that THOC5 does not play a role in export of a bulk polyA + RNA. In agreement with these data, Katahira et al. [29] and Chi et al. [30] showed that the depletion of THOC5 in Hela cells did not cause the accumulation of polyA + RNA in the nucleus. Similar data was also obtained using the HEK293 system [31]. Depletion of THOC5 however, causes severely altered phenotypes in mice, indicating that THOC5 is involved in indispensable functions for the differentiation process. These facts raise the question as to which mRNAs are THOC5 dependent.

**Figure 3:**
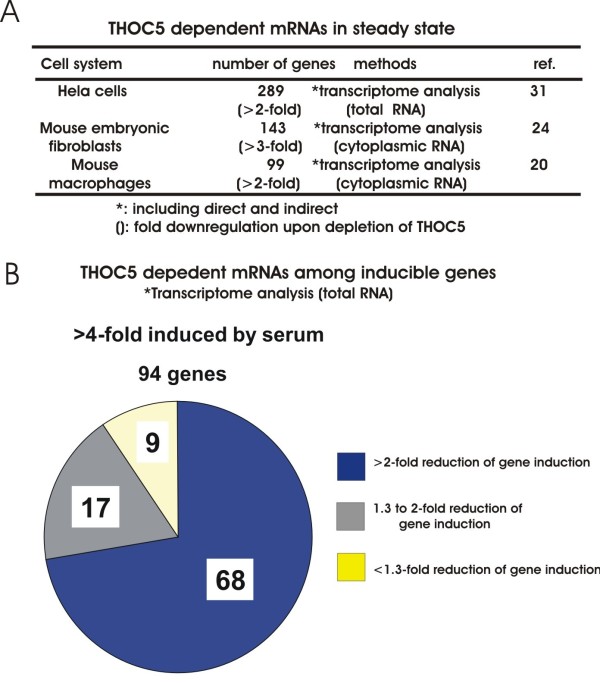
**Identification of THOC5 dependent genes in steady state and under serum stimulation conditions. A:** Summary table of transcriptome analyses in THOC5 depleted Hela cells [32], mouse embryonic fibroblasts [25], and bone marrow derived macrophages [20]. **B:** Transcriptome analysis of serum inducible genes in mouse embryonic fibroblasts from tamoxifen inducible THOC5 knockout mice [21]. Mouse embryonic fibroblasts with or without THOC5 were serum starved for 24 h and then stimulated with serum for 1 h. Total RNA was isolated for transcriptome analysis as described previously [20]. Ninety-four genes were upregulated more than 4-fold in the presence of THOC5. In absence of THOC5, only 9 genes were induced to a similar extent. * including direct and indirect THOC5 dependent genes.

THOC5 dependent mRNAs were studied by transcriptome analysis before or after depletion of THOC5 in three systems, namely human Hela cells, mouse embryonic fibroblasts, and mouse bone marrow derived macrophages (Figure 3A). In Hela cells the knockdown of THOC5 leads to down-regulation of 289 genes (>2-fold) [32]. In the mouse embryonic fibroblast system [25] and in the macrophage system [20] depletion of THOC5 caused the downregulation of 143 (>3-fold) and 99 (>2-fold) genes, respectively. The Ingenuity Pathway Analysis (IPA) of these genes reveals that over 60% of the genes were involved in differentiation/proliferation/motility. These included SRY-box containing gene (*Sox*) family genes, inhibitor of DNA binding (*ID*) family genes, Homeobox *(Hox)A1* or *HoxB3,* and polycomb gene *CBX2*[20,25] that are required for the maintenance of primitive cells.

## Extracellular stimuli induced mRNAs and THOC5

In the *yeast* system, it has been demonstrated that the THO complex plays a crucial role in processing of heat shock induced mRNA [33-35]. Recently, Mouaikel et al. [34] demonstrated that a high-frequency promoter associated with the THO complex function in the yeast system. Does THO complex in mammalian system also plays a role in highly inducible genes, such as immediate-early response genes?

When macrophages were stimulated with M-CSF, the expression of different sets of immediate-early response genes, such as *Ets* family genes, *Myc, Fos,* early growth response 1 *(Egr1),* or *JunB* are induced [36]. Indeed, *Ets* family genes failed to be upregulated upon stimulation with M-CSF in the absence of THOC5 [20]. Furthermore, Wnt (wingless/integrated) signal target genes, achaete-scute complex homolog 2 (*Ascl2), Sox9*, or snail family zinc finger (*Snai*)1, which play a key role in maintenance of intestinal stem cells and epithelial cell differentiation [37-42], are THOC5 dependent [21], indicating that the observed pathological alterations resulting from the depletion of THOC5 in intestinal epithelial cells are due to the lack of Wnt target gene expression.

Which population of inducible genes is indeed THOC5 dependent? Our recent data obtained from transcriptome analysis using serum induced mRNAs in mouse embryonic fibroblasts reveal that upon stimulation with serum, 94 genes were upregulated more than 4-fold within 1 h in the presence of THOC5. In THOC5 depleted cells, however only 9 genes were upregulated to a similar extent (Figure 3B), indicating that redundant backup pathways only partially rescue mRNA processing in the absence of THOC5 under serum induced conditions. These genes can be divided into two categories: One set of genes (e. g. *ets* family genes) [20] were not induced in the absence of THOC5. Another set of genes were induced in delayed manner, and spliced, but not exported (e. g. *Smad* family genes, *Snai1*) [20,21]. In agreement with these data, Grb10 interacting GYF protein 2 *(Gigyf2* or *Tnrc15),* and protein kinase C binding protein 1 *(Prkcbp1)* that were identified as THOC5 target genes using the fibroblast system were spliced but not exported in the absence of THOC5 [25]. These data imply that THOC5 plays a role in mRNA processing and export, however a redundant backup pathway may rescue the mRNA processing of a subset of genes, but not the export.

## Potential molecular function of THOC5 in immediate-early gene processing

**Figure 4:**
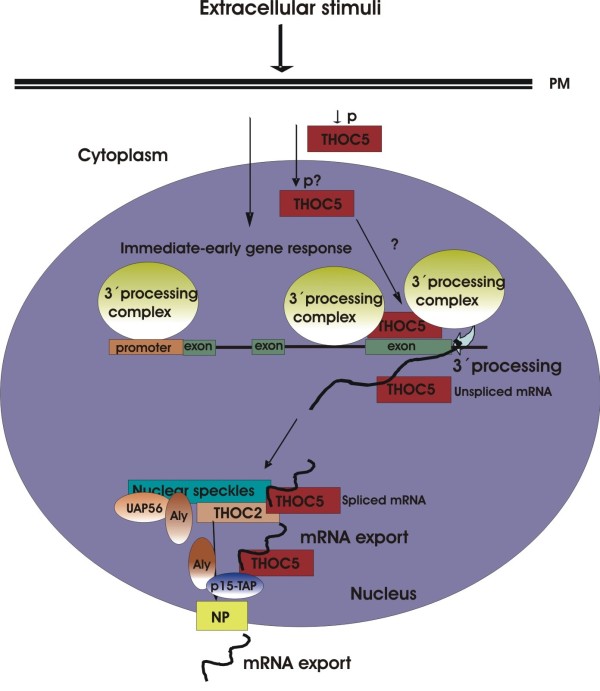
**Potential function of THOC5 in immediate-early gene response.** Upon stimulation with growth factors/cytokines THOC5 is phosphorylated at multiple positions. Signals reach the nucleus and the promoters of immediate-early response genes are activated. Simultaneously, the 3′ processing complex is recruited to the promoter region [32]. During gene transcription, THOC5 is recruited to its target immediate-early gene [20] and then participates in 3′ processing of these genes. Unspliced and spliced THOC5 target mRNAs form a complex with THOC5. Post transcriptional splicing occurs in nuclear speckles where THOC5 has accumulated [20]. For the export of spliced mRNAs from nuclear speckles other members of TREX, Aly, UAP56 and THOC2 are required [30,44]. It has been suggested that THOC5 and Aly form a complex with mRNA receptor TAP-p15 transiently and transfer THOC5 target mRNA to the TAP-p15 complex [29]. PM: plasma membrane; NP: nuclear pore.

Lipopolysaccharide induced full-length nascent RNAs that contain introns accumulated on chromatin prior to release of functional mRNA for export [43]. These RNAs are post-transcriptionally spliced. Our preliminary data reveal that in the absence of THOC5 chromatin associated *Ets1* mRNA accumulated to a greater extent than in the presence of THOC5 after M-CSF stimulation. These data suggest that THOC5 may play a role in release of its target immediate-early gene transcripts from chromatin. In agreement with these data, Katahira et al. recently showed that THOC5 plays a role in 3′ processing of THOC5 target genes in Hela cells [32]. The same authors show that THOC5 interacts with polyadenylation specific factor 6, (CPSF6, or CFIm68). Moreover, CFIm68 is not properly recruited to the 5′ end of certain genes in THOC5 depleted cells and that in these genes 3′end cleavage is altered. CPSF6 is recruited to the promoter region of THOC5 target genes, however it is not clear to where THOC5 is recruited in this system [32]. We recently showed that THOC5 is recruited to the last exon, but not the promoter region of *Ets1* gene upon stimulation with M-CSF [20] (Figure 4). Thus, it could be that in a subset of genes, THOC5 dictates the 3′ cleavage site by directly recruiting the CPSF machinery, while in other transcripts, such as *Ets1*, it does not.

The mechanism by which THOC5 is recruited to its target genes and how the depletion of THOC5 reduces the recruitment of the 3′ processing complex still remains to be studied. It has been shown that unspliced and spliced *Ets1* mRNA bind to THOC5 [20], and THOC5 is mainly detected in nuclear speckles where post-transcriptional splicing occurs [44-46], suggesting that THOC5 may be involved in mRNA localization in nuclear speckles. In addition, it has been documented that for the export of spliced mRNAs from nuclear speckles, other members of TREX, Aly, UAP56 and THOC2 are required [30,44]. It has been suggested that THOC5 and the adapter protein Aly concomitantly bind the mRNA receptor Tap-p15 and play a role in Tap-p15 mediated nuclear export of *Hsp70* mRNA under heat shock conditions [29] (Figure 4). However, the exact role of THOC5 in 3′ processing, and export of growth factor/cytokine inducible genes still remains to be studied.

## Target selectivity of THOC5

How does THOC5 select its target mRNAs? Interestingly, all rapidly-inducible genes, such as *Fos, Egr1,* immediate-early response 2 (*Ier2*), or *JunB* which are identified in macrophages stimulated by lipopolysaccharides [43] are induced and exported without alteration in THOC5 depleted cells [20]. These are intron-less (*Jun, Ier2*) or co-transcriptionally spliced (*Fos, Egr1*) genes. THOC5 dependent immediate-early genes which have so far been indentified are spliced post-transcriptionally. Recently, Katahira et al. [32] proposed that THOC5 controls an alternative polyadenylation site choice by recruiting cleavage factor CPSF6 on target genes. However, 70-75% of human mRNAs contain a potential alternative polyadenylation site [47], suggesting that THOC5 dependency may also be associated with different RNA element(s). It has been previously shown in the yeast system that the yeast homolog of THOC1, Hpr1 is required for transcription of either long or GC rich DNA sequences [2]. Interestingly, the coding regions (not in the 3′-UTR) of *Sox9*, *Ascl2*, *Snai1*, and *Wnt11* (THOC5 dependent genes) contain high percentages of GC degree (61-67%), while THOC5 independent genes contain 54-55% GC [21]. However, GC-content is known to vary even within most genes [48]. Furthermore, the target selectivity of THOC5 is highly complex, because redundant backup pathways partially rescue mRNA 3′ processing and export to a different degree in the absence of THOC5. Identifying the binding partners of THOC5 for the 3′ processing or the export of its target mRNAs, and further identification of THOC5 target genes may provide more insight into its target selectivity.

## Potential applications

The findings described above suggest that the level of THOC5 in cells is one of key factors for controlling the immediate-early gene response. We have previously shown that a 50% reduction of endogenous THOC5 in C2C12 cells causes adipocyte differentiation under muscle differentiation conditions, while a 2-fold over-expression of THOC5 causes muscle differentiation under adipocyte differentiation conditions [22]. In agreement with these conditions for generating phenotypes, CCAAT/enhancer-binding protein (*C/EBP*) family genes (no intron) that play a key role in adipocyte differentiation are THOC5 independent (GEO series accession number GSE41170), while *Sox* family genes, *CBX2, ID2* or *HOXB3* that participate in muscle differentiation are THOC5 dependent [20,25]. This mechanism may also served as a useful tool to manipulate lineage commitments from mesenchymal stem cells by down- or upregulation of THOC5 expression at different levels.

Furthermore, the knockdown of THOC1 or THOC5 did not cause apoptosis in normal cells, but it causes apoptosis in cancer cells [25,27,49]. In addition, recent data imply the involvement of THOC5 in leukemia development [13]. These data suggest that members of THO complex could be a target molecule for cancer therapy.

## Conclusions

THOC5 that couples transcription and mRNA processing with mRNA export is a key molecule for maintenance of stem cells and for differentiation/proliferation in multiple organs. THOC5 is required for the 3′ processing and/or export of most cytokine/growth factor induced genes. Thus, THOC5 is one of key factors to commit cell lineage and to mediate fine tuning during cell differentiation. Presently, however understanding of the molecular function of THOC5 in immediate-early gene response is still in its infancy. Does phosphorylation influence its function and cancer development? How is THOC5 recruited to active immediate-early genes? How does THOC5 select its target genes? Does THOC5 participate in post-transcriptional splicing? These questions remain to be answered.

## Competing interests

The authors declare that they have no competing interests.

## Authors’ contributions

DDHT, AK and TT drafted and wrote the manuscript. All authors participated in the discussion and approved the final manuscript.
